# Deconvolution of heterogeneous tumor samples using partial reference signals

**DOI:** 10.1371/journal.pcbi.1008452

**Published:** 2020-11-30

**Authors:** Yufang Qin, Weiwei Zhang, Xiaoqiang Sun, Siwei Nan, Nana Wei, Hua-Jun Wu, Xiaoqi Zheng

**Affiliations:** 1 College of Information Technology, Shanghai Ocean University, Shanghai, China; 2 Key Laboratory of Fisheries Information Ministry of Agriculture, Shanghai, China; 3 School of Science, East China University of Technology, Nanchang, Jiangxi, China; 4 Zhongshan School of Medicine, Sun Yat-Sen University, Guangzhou, China; 5 Department of Mathematics, Shanghai Normal University, Shanghai, China; 6 Department of Biostatistics and Computational Biology, Dana-Farber Cancer Institute and Harvard School of Public Health, Boston, Massachusetts, United States of America; University of Michigan, UNITED STATES

## Abstract

Deconvolution of heterogeneous bulk tumor samples into distinct cellular populations is an important yet challenging problem, particularly when only partial references are available. A common approach to dealing with this problem is to deconvolve the mixed signals using available references and leverage the remaining signal as a new cell component. However, as indicated in our simulation, such an approach tends to over-estimate the proportions of known cell types and fails to detect novel cell types. Here, we propose PREDE, a partial reference-based deconvolution method using an iterative non-negative matrix factorization algorithm. Our method is verified to be effective in estimating cell proportions and expression profiles of unknown cell types based on simulated datasets at a variety of parameter settings. Applying our method to TCGA tumor samples, we found that proportions of pure cancer cells better indicate different subtypes of tumor samples. We also detected several cell types for each cancer type whose proportions successfully predicted patient survival. Our method makes a significant contribution to deconvolution of heterogeneous tumor samples and could be widely applied to varieties of high throughput bulk data. PREDE is implemented in R and is freely available from GitHub (https://xiaoqizheng.github.io/PREDE).

## Author summary

Tumor tissues are mixtures of different cell types. Identification and quantification of constitutional cell types within tumor tissues are important tasks in cancer research. The problem can be readily solved using regression-based methods if reference signals are available. But in most clinical applications, only partial references are available, which significantly reduces the deconvolution accuracy of the existing regression-based methods. In this paper, we propose a partial-reference based deconvolution model, PREDE, integrating the non-negative matrix factorization framework with an iterative optimization strategy. We conducted comprehensive evaluations for PREDE using both simulation and real data analyses, demonstrating better performance of our method than other existing methods.

This is a *PLOS Computational Biology* Methods paper.

## Introduction

Tumor tissues are heterogeneous and consist of different cell types including tumor cells (or sub-clones) and various microenvironmental cell types such as infiltrating immune cells and stromal cells [1–3]. The intra-tumor heterogeneity is reported to be closely related to clinical outcomes such as tumor growth, metastasis, recurrence and drug resistance [4]. Therefore, it is of great significance to accurately quantify the degree of tumor heterogeneity, including the number of cell populations contained in tumor tissues, the molecular profile of each cell population and their proportions.

With the rapid development of high-throughput sequencing technology, a large number of genome, epigenome, transcriptome and proteome data of tumor samples have been profiled. Such biomedical big data provide a possibility to study tumor heterogeneity from the molecular perspective by using computational methods. Although the recent emerging single-cell sequencing technology strives to tackle these problems by measuring expression profiles of thousands to millions of cells simultaneously, it is yet not feasible to be conducted for large cohort studies due to, for example, expensive cost and extensive dropout events [5]. Therefore, quantification of tumor heterogeneity from the bulk omics data is profoundly important, particularly in clinical situations.

In recent years, many computational methods have been proposed for bulk data deconvolution [6–10]. At present, these methods can be roughly divided into two categories: reference-based methods [9,11], and reference-free methods [12–14]. The first type of methods requires cell type-specific gene expressions (i.e., reference) as input, and the proportion of each cell type can be analyzed by constrained projection algorithms such as constrained linear regression or support vector regression. However, for many practical reasons, it is virtually impossible to obtain gene expression profiles of all cellular components in tumor tissues [15]. As such, reference-based methods are only applicable for special diseases such as blood or brain cancers or only focus on specific cell types such as immune cells [10,16], where major cellular components are clear and reference signals are available [17]. The second type of methods does not rely on reference information and aims to estimate molecular profiles and compositions of all cell types simultaneously [6,12,13,18,19]. Although these methods do not require cell-type expression profiles as input, they rely on known cell-type proportions as prior information [8,20,21].

However, in real clinical practice, only a fraction of cell types is known while the rest are unknown so the deconvolution problem should be subject to partial reference. A straightforward way to deal with this problem is to use current available information of known cell types as the reference to deconvolve the whole mixture signals, or assume all unknown proportion to be from one cell type [22]. However, as will be illustrated in our simulation section, such a strategy fails to account for new cell types, and is prone to overestimate proportions of known cell types.

In this paper, we proposed a *p*artial-*re*ference based *de*convolution (PREDE) model based on the non-negative matrix factorization (NMF) framework using an iterative optimization strategy to address the above challenges. Using the expression profiles of the available cell types as input, PREDE could simultaneously estimate both the proportions of all cell types and the expression profiles of unknown cell types. We performed comprehensive evaluations for the proposed deconvolution method in comparison with other existing methods using both simulated data and real dataset of tumor samples. The results demonstrated that PREDE could effectively deconvolve mixture tumor samples from partial reference signals and could reveal novel insights into tumor heterogeneity and clinical prognosis.

## Results

### Overview of PREDE

As the previous methods did [23], we assume that the considered heterogeneous samples compose of fixed number of cell types whose expression profiles are relatively stable across samples [24]. The deconvolution problem is usually formularized to *Y* = *WH*+*ϵ*, where *Y* represents expression matrix of heterogeneous samples, *W* is basis matrix representing the quantitative expression profiles of constitutional cell types and *H* is the proportion matrix. If quantitative profiles of cell types (i.e. *W*) are known, it is so-called reference-based deconvolution. Alternatively, if both basis matrix *W* and proportion matrix *H* are unknown, it is so-called reference-free deconvolution.

**Fig 1:**
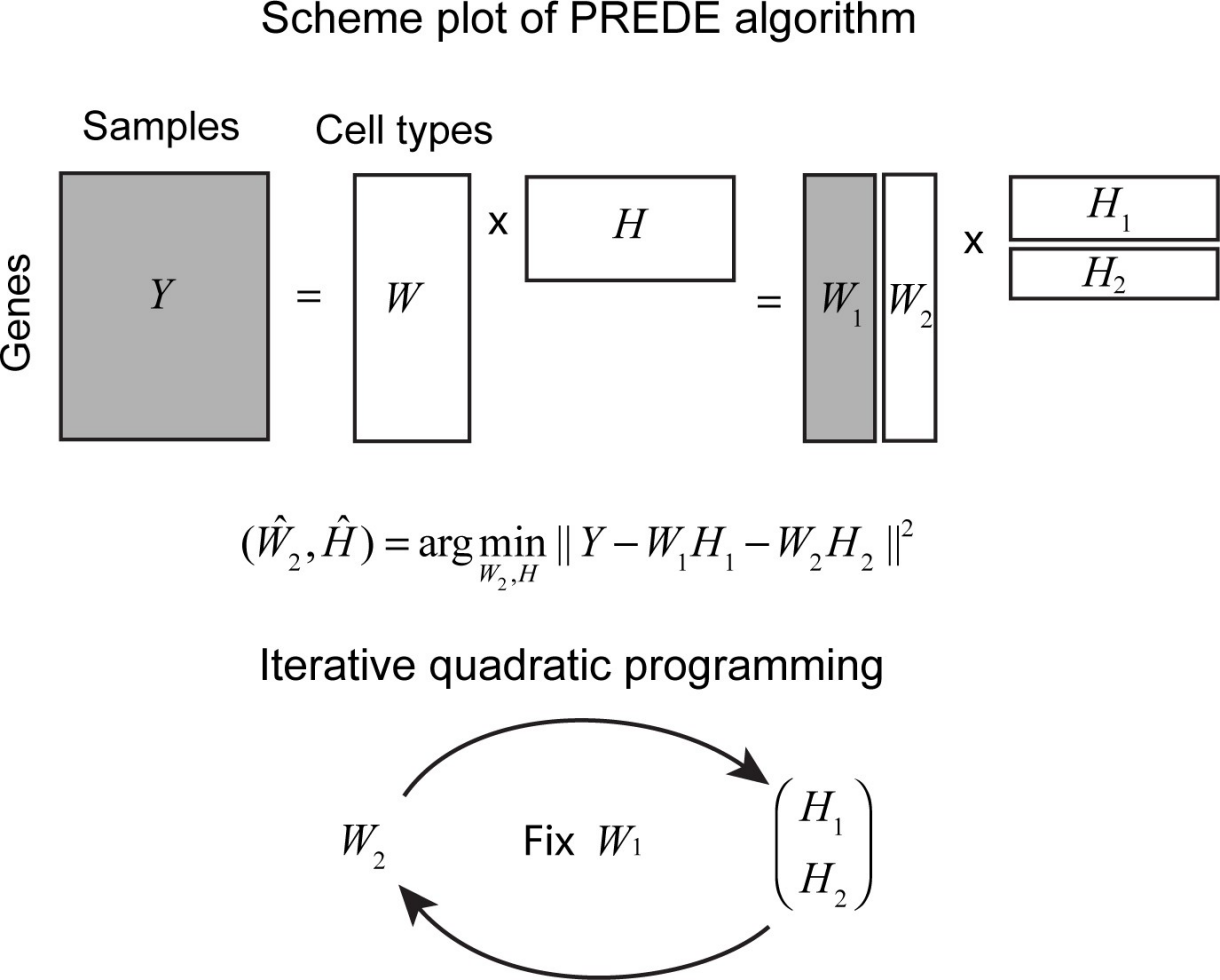
The workflow of partial reference-based deconvolution (PREDE). Given expression matrix of heterogeneous samples *Y* and known reference matrix *W*_1_, PREDE aims to infer the proportion matrix *H* for all constituent cell types and expression matrix for unknown part *W*_2_. The deconvolution problem is formulated to an NMF model which is solved via an iterative Quadratic Programming procedure by fixing *W*_1_ in each iteration.

In real clinical practice, only a fraction of the cell types in tumor samples is available. We denote the available portion of basis matrix *W* as *W*_1_, and the unknown portion as *W*_2_, i.e., *W* = (*W*_1_, *W*_2_). Given expression matrix *Y* for all tumor samples and basis matrix *W*_1_, PREDE infers the basis matrix *W*_2_ for unknown cell types and overall proportion matrix *H*. The main workflow of PREDE is briefly illustrated in Fig 1. We solve the above problem by iteratively applying the constraint Quadratic Programming algorithm until convergence, with *W*_1_ fixed in each iteration (see Materials and Methods for detail).

Our PREDE method can be viewed as a generalization of previous reference-based and reference-free deconvolution algorithms. If known reference *W*_1_ are complete (i.e., *W*_1_ = *W*), PREDE is actually the typical reference-based deconvolution method. On the other hand, if expression profile of any cell type is unavailable (i.e., *W*_1_ is null), PREDE then becomes the typical reference-free method.

### Benchmarking PREDE with cell line mixture data

We conducted a series of simulations to comprehensively evaluate the performance of PREDE, by considering three factors in the simulations: noise ratio, expression similarity between cell lines and proportion of rare cell types. To this end, we downloaded gene expression profiles of 91 lung cancer cell lines from the CCLE dataset and selected some of them as reference *W*. Gene expression matrix *Y* for mixture samples is then obtained by multiplying *W* with a randomly proportion matrix *H* generated from the Dirichlet distribution, followed by an additional error matrix with Gaussian distribution. *Y* and available reference matrix *W*_1_ were used as inputs of PREDE and Akaike information criterion (AIC_c_) was employed to determine the optimal number of cell types.

**Fig 2:**
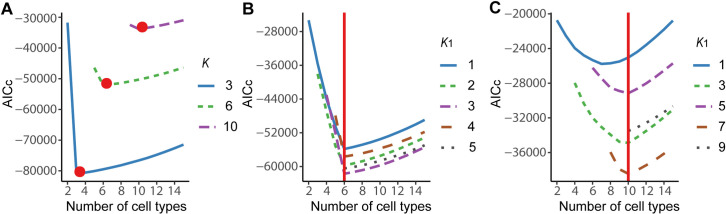
Accuracy of AIC_c_ in identifying the number of cell types using simulated data. (A) AIC_c_ values when the total number of cell types is 3, 6 or 10, but only 1, 4 or 8 cell types are known, respectively. (B) AIC_c_ values when 1, 2, 3, 4 or 5 of total 6 cell types are known. (C) AIC_c_ values when the number of total cell types is 10 and the numbers of known cell types are 1, 3, 5, 7 and 9, respectively.

We first evaluated the accuracy of AIC_c_ in determining the number of cell types from mixture samples. Following the above simulation procedure, we randomly selected 3, 6 and 10 lung cancer cell lines to generate 100 mixture samples respectively, and assumed only a fraction of cell lines to be known. Fig 2A shows the AIC_c_ scores when *W* consists of 3, 6 and 10 cell types, but only 1, 4 and 8 of them are supposed to be known. As expected, AIC_c_ decreased first and then gradually increased with the increase of numbers of cell types, and correctly achieved the minimum values at *K* = 3, 6 and 10 respectively. We further investigated the accuracy of AIC_c_ in determining the total number of cell types when different numbers of known cell types were used for the input. When the total number of cell types was 6, AIC_c_ successfully reaches the minimum value at *K* = 6, regardless of the numbers of known cell types (Fig 2B). But when the total number of cell types increased to 10 and only a small number of cell types was known (e.g., *K*_1_ = 1), the predicted number of cell types is slightly underestimated (Fig 2C).

**Fig 3:**
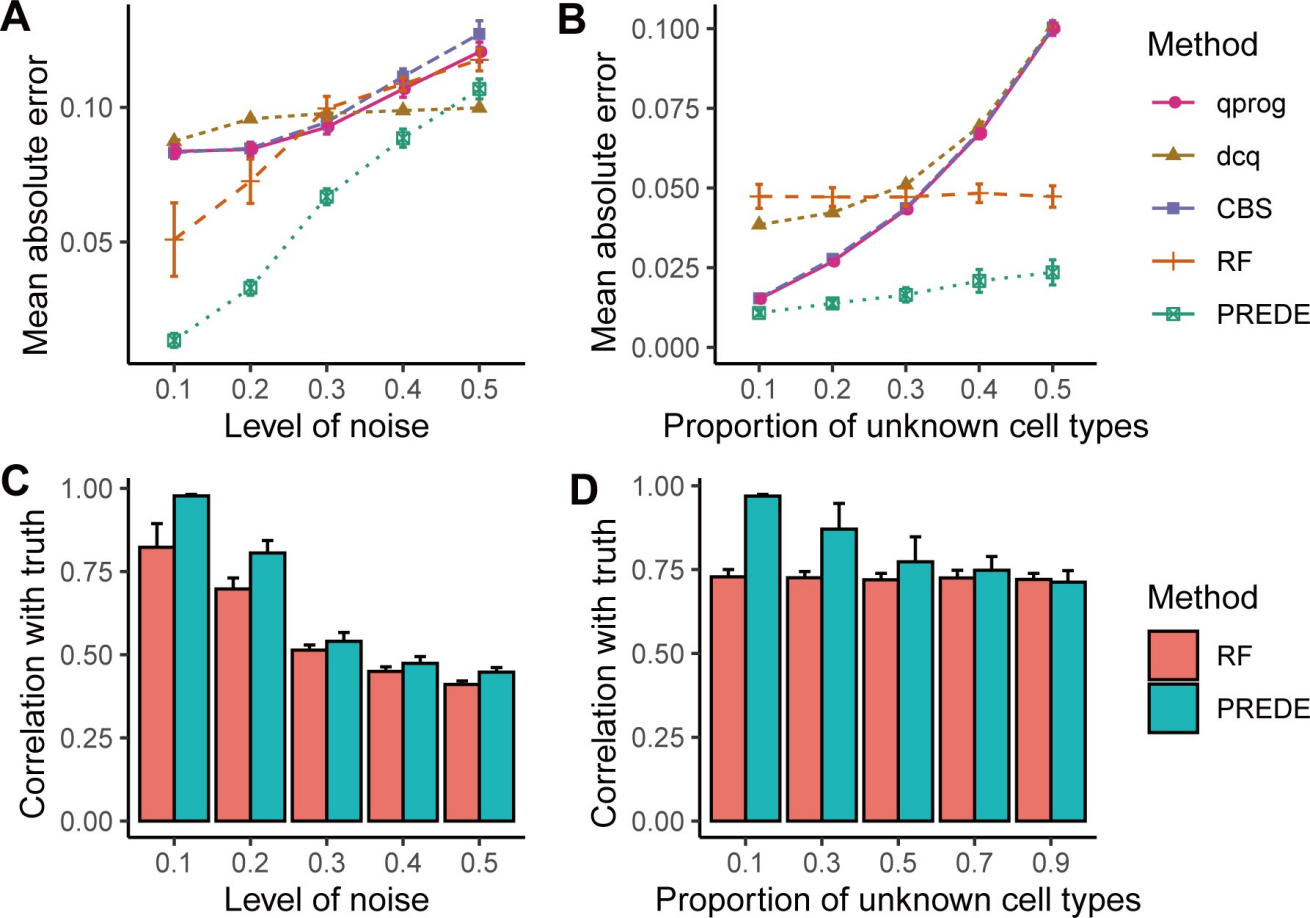
Comparing different deconvolution methods for estimating cellular proportions and expression profiles from mixture data. (A-B) Mean absolute errors between the true and predicted cellular proportions by four methods from the simulated data with different levels of noise (A) or different proportions of unknown cell types (B). (C-D) Correlations between true and predicted expression profiles of unknown cell types by PREDE or reference-free methods under different levels of noise (C) or proportions of unknown cell types (D). All simulations were repeated 20 times.

Based on the above simulation datasets, we compared our PREDE method with four existing methods, i.e., qprog (constrained linear regression solved by quadratic programing) [25], dcq (digital cell quantification using elastic net regularization) [26], CIBERSORT (CBS, state-of-the-art tool for inferring tumor-infiltrating immune cells using support vector regression) [10], and a reference-free deconvolution using NMF (RF) [19]. Two iterative methods, i.e., RF and PREDE, adopted the same condition for convergence. CIBERSORT was implement by the ‘svm’ function from the e1071 package, where the hyperparameter *μ* is optimally selected by cross-validation. All above methods take the top 1000 genes with the largest coefficient of variation as input. We calculated the mean absolute error (MAE) between true and predicted proportions of available cell types for all four methods at different levels of noise (Gaussian distributions with mean 0 and standard deviation of *c*×*m*, where *c* ranges from 0.1 to 0.5 with step 0.1, *m* is the mean expression for each gene in mixing samples). PREDE obtained the lowest biases and relatively stable results at all levels of noise, compared to qprog, dcq and CIBERSORT (Fig 3A). In addition, we evaluated the performance of four methods in estimating cell-type proportions when unknown cell fractions increase from 0.1 to 0.5 (Fig 3B). Our method also showed constantly the lowest MAE at different unknown fractions, especially when unknown fractions exceed 0.2. Similar conclusion can be drawn when using Pearson correlation coefficients between true and predicted cell proportions as measurement for proportion estimation (S1 Fig).

Besides proportion estimation, our partial reference deconvolution method (as well as RF) is also capable of inferring gene expression profiles of unknown cell types. Fig 3C and 3D show correlation coefficients between predicted and true expression profiles derived from the two methods at different levels of noise (Fig 3C) and unknown cell fractions (Fig 3D). Our method exhibited consistently higher accuracy compared to the reference-free method.

We also evaluated the performance of our method when one unknown cell type is highly similar to known cell types. To this end, we constructed two simulation datasets by selecting different sets of lung cancer cell lines from the CCLE dataset. The first is ‘low similarity set’, which consists of 6 cell lines (4 known and 2 unknown) with relatively low Pearson correlation coefficient (PCC) (0.75~0.8) between each pair of cell lines. The second is ‘high similarity set’, which also consists of 4 known and 2 unknown cell types but one unknown cell line is highly correlated with a known one (with PCC about 0.95). For both datasets, PREDE and RF were used to infer proportions of unknown cell lines, where prediction accuracies were measured by MAE and Pearson correlations between predicted and true proportions. We found that for both criteria, PREDE showed relatively lower biases and higher Pearson correlations in recovering the proportions of unknown cell type compared with RF (S2 Fig).

We then evaluated the performance of PREDE on recovering rare populations that may be of biological importance. We mixed tumor samples from the 6 CCLE lung cell lines including one rare type with proportion varying from 0.01 to 0.15. We first examined whether AICc could infer the number of total cell types from the mixture samples. When proportion of the rare cell type was small (e.g., less than 0.05), AICc achieved its minimal value at *K* = 5 (S3 Fig). This indicates that when the proportion of a cell type was too small, AICc failed to recognize it as an independent cell type but treated it as noise or merged it into other major cell types. But if its proportion was moderately large, i.e., exceeds 0.07, AICc could successfully identify the total number of cell types (*K* = 6). Then we sought to evaluate the performance of the PREDE and RF in inferring proportion and expression profile of the *unknown* rare population when *K* was given. PREDE showed a consistently lower proportion bias and higher profile correlation than RF when proportions of the rare cell type changed from 0.07 to 0.10 (S4 Fig). Similarly, we also performed simulations for the situation that the proportion of a *known* cell type was rare in the total mixture. Our method also showed constantly the lowest MAE compared with three other methods (S5 Fig).

### Estimation of immune and cancer cell expression and proportion from cell line mixtures

**Fig 4:**
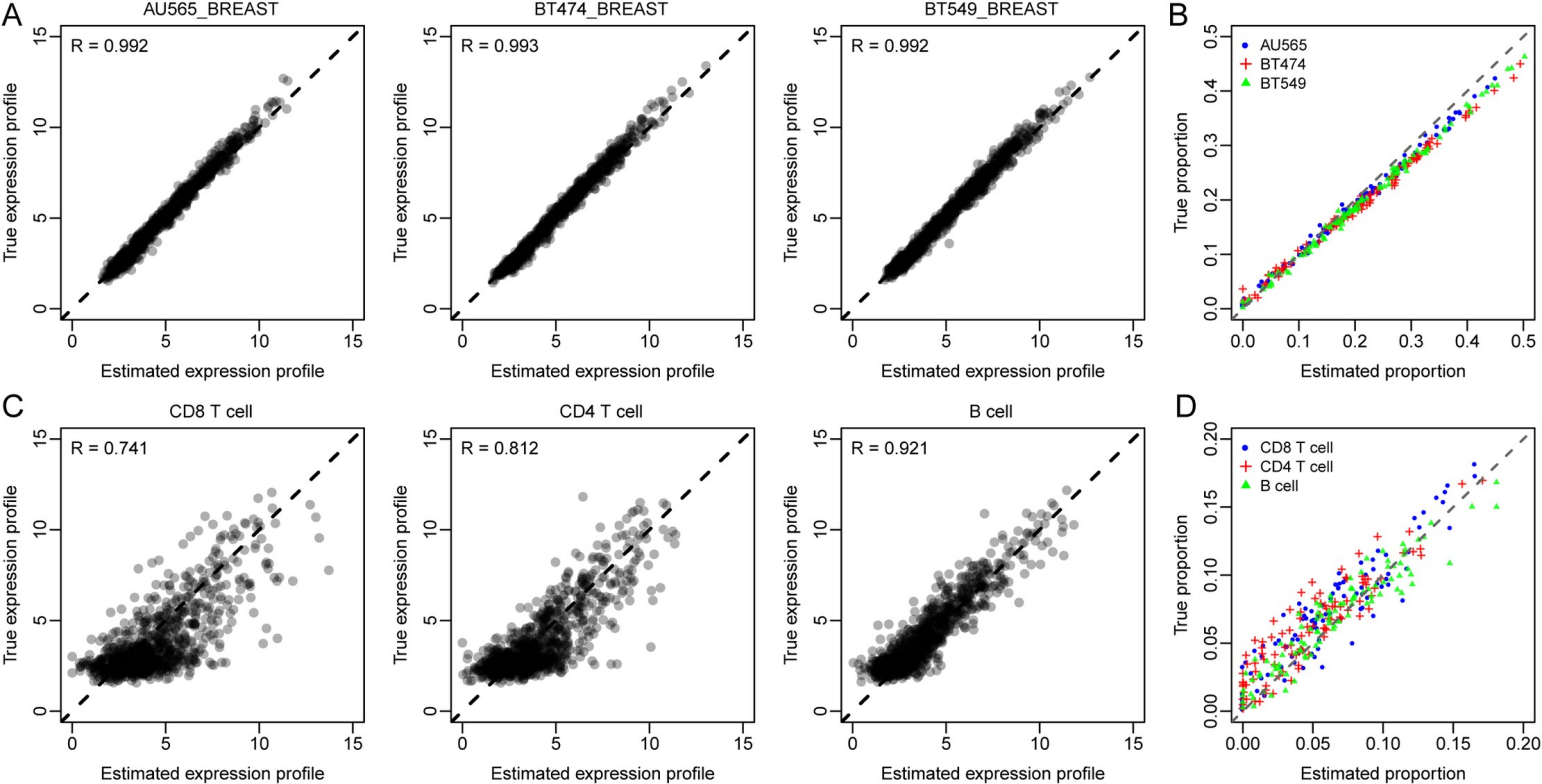
Estimating expression profiles and cellular proportions of cancer cells and immune cells using PREDE. Eight cell lines including three breast cancer cell lines, three immune cell lines, and two normal cell lines were mixed with proportions 60%, 20%, and 20% respectively to mimic tumor immune microenvironment. Shown are accuracies of profiles and proportion estimations when (A-B) expressions of cancer cells are missing and (C-D) expressions of immune cells are missing.

We next tested our method in a situation that is more relevant to cancer immunology study. Gene expressions of 8 cell lines (including 3 breast cancer cell lines, 3 immune cell lines and 2 normal cell lines, see Method section for details) were mixed together to simulate 100 tumor samples with roughly 60% cancer cells, 20% immune and 20% normal cells for each sample. We considered the following two scenarios of the deconvolution: i) expressions of three types of tumor cells are unavailable; ii) expressions of immune cells are unavailable. AIC_c_ curves (S6 Fig) showed that our method correctly predicts the total number of cell types (i.e., 8) for both two scenarios based on the mixture data. We then used PREDE to infer the proportions and expression profiles of the unknown cell types. PREDE could correctly recover the expression profiles of missing cell types and their respective proportions in tumor samples (Fig 4). For comparison, we also applied reference-free deconvolution method to the mixed samples (S7 Fig). Given the true number of cell types as input, the reference-free deconvolution method resulted in much lower accuracies in profile and proportion estimations than PREDE, in consistent with the above results (Fig 3).

### Validation of PREDE using rat tissues mixture data

**Fig 5:**
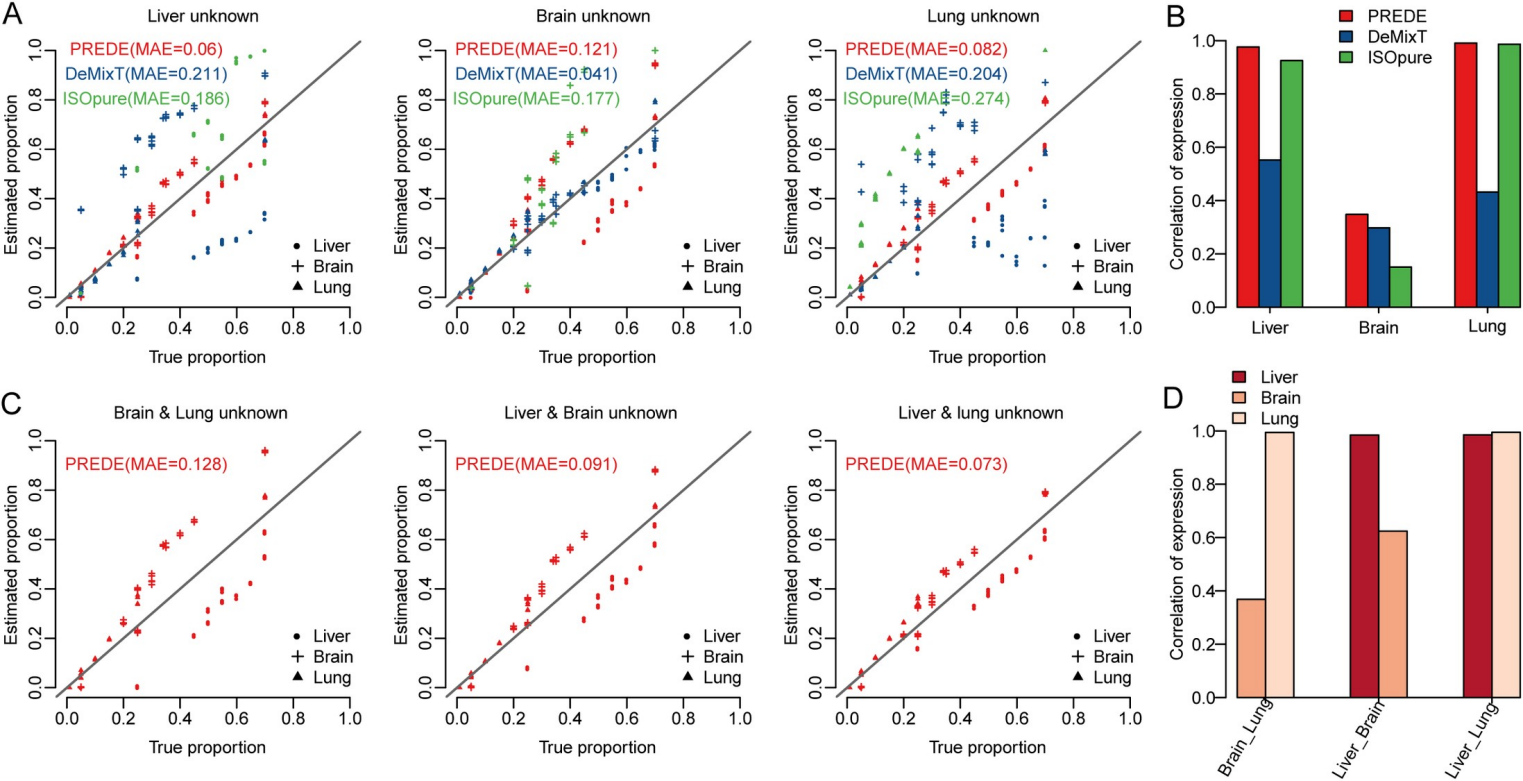
Application of PREDE to rat tissue mixture data. (A) Estimated proportions of three tissue types by PREDE, DeMixT, and ISOpure when one tissue is unknown, in comparison with true proportion. The mean absolute error (MAE) between true and predicted proportions is used to evaluate the accuracy of the proportion estimation. (B) Correlations between true and predicted expression profiles of unknown cell types when one tissue is unknown. (C) Estimated proportions of three tissue types by PREDE when two of three tissue types are unknown, in comparison with true proportions. (D) Correlations between true and predicted gene expression profiles of unknown tissues by PREDE when two of three tissue types are unknown.

To better mimic the real biological scenario, we further evaluated our method on a gene expression dataset [21] consisting 30 samples mixed from liver, brain and lung tissues that were derived from a rat with known proportions. We evaluated our method under the following two scenarios: 1) one of three tissues (brain, liver, and lung) was assumed to be unknown (Fig 5A and 5B); 2) two of three tissues were assumed to be unknown (Fig 5C and 5D).

We compared our method with two state-of-the-art methods for the same problem, i.e., DeMixT [27] and ISOpure [28]. DeMixT is a three-component statistical model for the deconvolution of tumor sample heterogeneity, an updated version of the previously developed DeMix [29]. ISOpure is a two-step statistical model to estimate tumor purities and individual cancer profiles using tumor mixture profiles and normal profiles as input. Note that DeMixT and ISOpure can estimate the profile of the remaining one cell type when expression profiles of *K*-1 cell types are available (*K* is the total number of cell components). If the number of known cell types is less than *K*-1, they will treat the remaining cell types (>1) as a merged single cell type. Thus, both DeMixT and ISOpure work when only one tissue in the above rat tissue mixture data is unknown (i.e., scenario 1), but only PREDE works even when two tissues are unknown (i.e., both scenarios 1 and 2).

When the profile of one tissue was assumed to be unknown, PREDE outperformed DeMixT and ISOpure in proportion estimation in the case that liver or lung profile was unknown (Fig 5A) and in profile estimations under all the three conditions (Fig 5B). In the case that two of the three tissue profiles were unknown, DeMixT and ISOpure were not applicable as mentioned above, while our PREDE method still got favorable results (Fig 5C). Fig 5D shows the prediction of expression profiles when two tissue types (i.e., brain & lung, liver & brain, as well as liver & lung) were unknown. Overall, our method exhibited robust and improved performance in terms of both tissue proportion and expression profiles estimations based on the rat tissue mixture data.

Since DeMixT and ISOpure are designed specifically for tumor tissue deconvolution, in addition to rat-tissue mixture data, we further evaluated DeMixT, ISOpure and our method based on a synthetic dataset used in Fig 4, i.e., 100 mixture samples mixed from 3 cancer cell lines, 2 normal cell lines and 3 immune cell lines. In our evaluation, each of the three cell types was assumed to be homogenous for applying DeMixT and ISOpure. Mean expression profiles of one cell types (for example, cancer cells) were treated as unknown cellular components and expression profiles of the rest two cell types (i.e., normal cells and immune cells) were used as input for all the three methods. S8 Fig shows the estimations of cellular proportion and expression profile for the unknown cellular component by all methods. We found that PREDE exhibited overall lower MAEs (S8A Fig) in proportion estimation and higher correlations (S8B Fig) with true profiles for unknown cell components.

### Validation of PREDE using PBMC samples

We further evaluated our method on a gene expression dataset of PBMC samples (*n* = 20) downloaded from the GEO database (GSE65136) where the corresponding flow cytometry measurement of proportions are available [10]. In deconvolution of the mixture PBMC samples, expression profiles of nine cell types from LM22 matrix are chosen as reference (*W*), and 3, 5 and 7 of them are selected as the known reference *W*_1_ to test PREDE as well as four other immune cell deconvolution tools, i.e., RF, CIBERSORT [10], dcq [26] and qprog [25]. Note that TIMER is designed specifically for estimating the abundance of six tumor-infiltrating immune cell types (B cells, CD4 T cells, CD8 T cells, neutrophils, macrophages, and dendritic cells) [16]. It does not accept subset of references as input, thus cannot ensure a fair comparison. Therefore, TIMER was not included in the comparison. Cell proportions measured by the flow cytometry were used as ground-truth to benchmark all the four methods.

**Fig 6:**
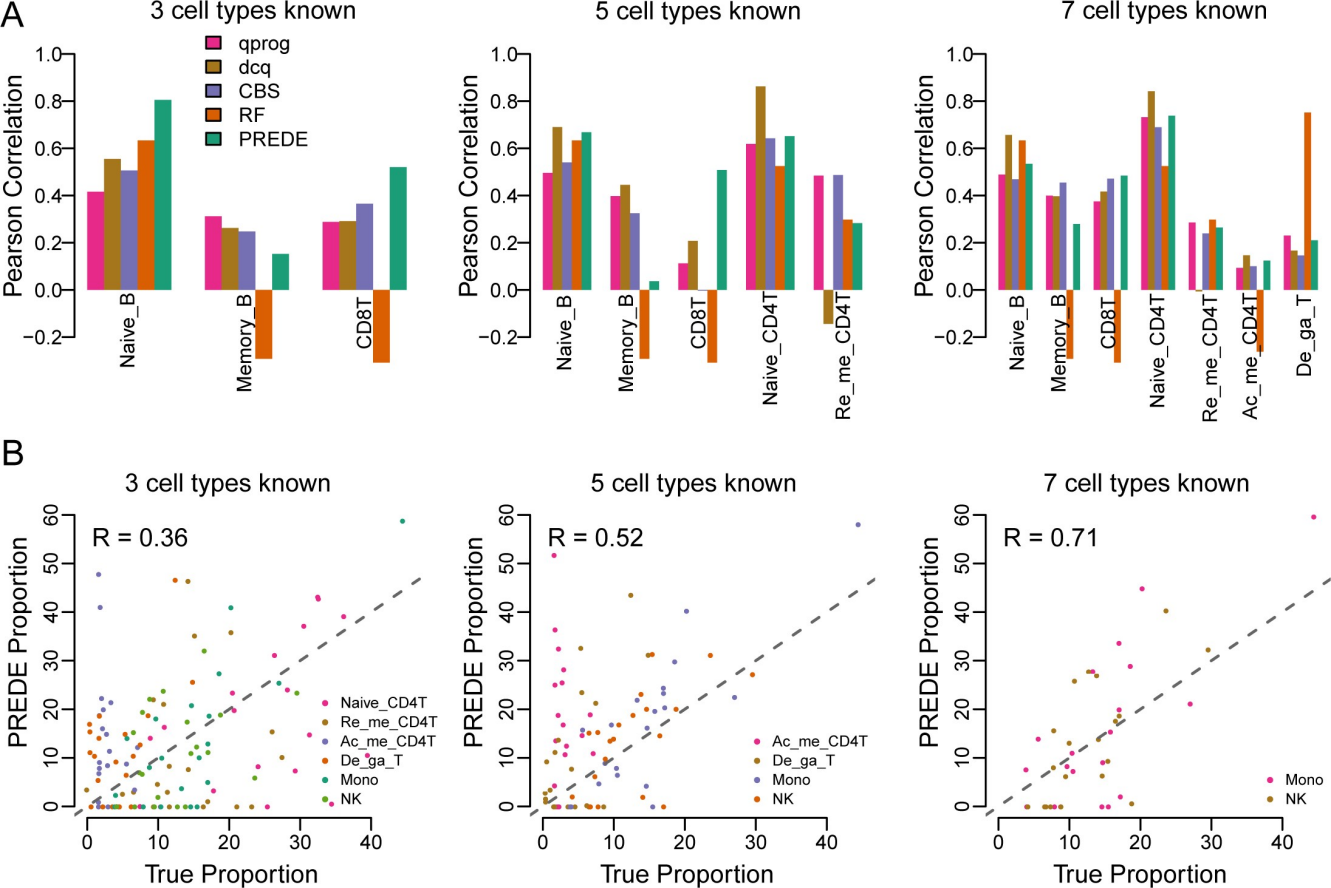
Application of PREDE to the PBMCs dataset. Deconvolution results by PREDE, RF, CIBERSORT, dcq and qprog when 3, 5 and 7 of 9 cell types were selected as the known reference. (A) Pearson correlations between estimated proportions by four methods and flow cytometry fractions for each known cell type. (B) Accuracies of PREDE in terms of proportion estimations for unknown cell types.

The Pearson correlation coefficients (PCCs) between the predicted and the true proportions were shown in Fig 6A. Overall, PREDE outperformed the other three methods when a small number of cell types were available. For example, when 3 cell types were known (Fig 6A, left panel), the PCCs between the true and the predicted proportions by RF, CIBERSORT, qprog and dcq for Naïve_B were 0.64, 0.51, 0.42 and 0.56, respectively, but that by PREDE achieved 0.81. When number of known cell types increased to 5 and 7, PREDE showed comparable (or even slightly better in some cases) performance with the other three methods. This is anticipated because when more cell components are known, PREDE is generally identical to the reference-based methods, as mentioned above.

Another superiority of PREDE is the ability to infer proportions of unknown cell types (Fig 6B). When only 3 cell types are known, the correlations between proportions estimated by PREDE and flow cytometry measurement is 0.8 for Mono, 0.55 for De_ga_T, and mean correlation for the remaining 6 unknown cell types is 0.36. When the number of known cell types increased from 5 to 7, the mean correlation increased from 0.52 to 0.71. All above results confirmed that PREDE could not only infer the proportions of known cell types, but also satisfactorily identify expression profiles of unknown cell types.

### Applications of PREDE to BRCA, SKCM and BLCA samples in TCGA

**Fig 7:**
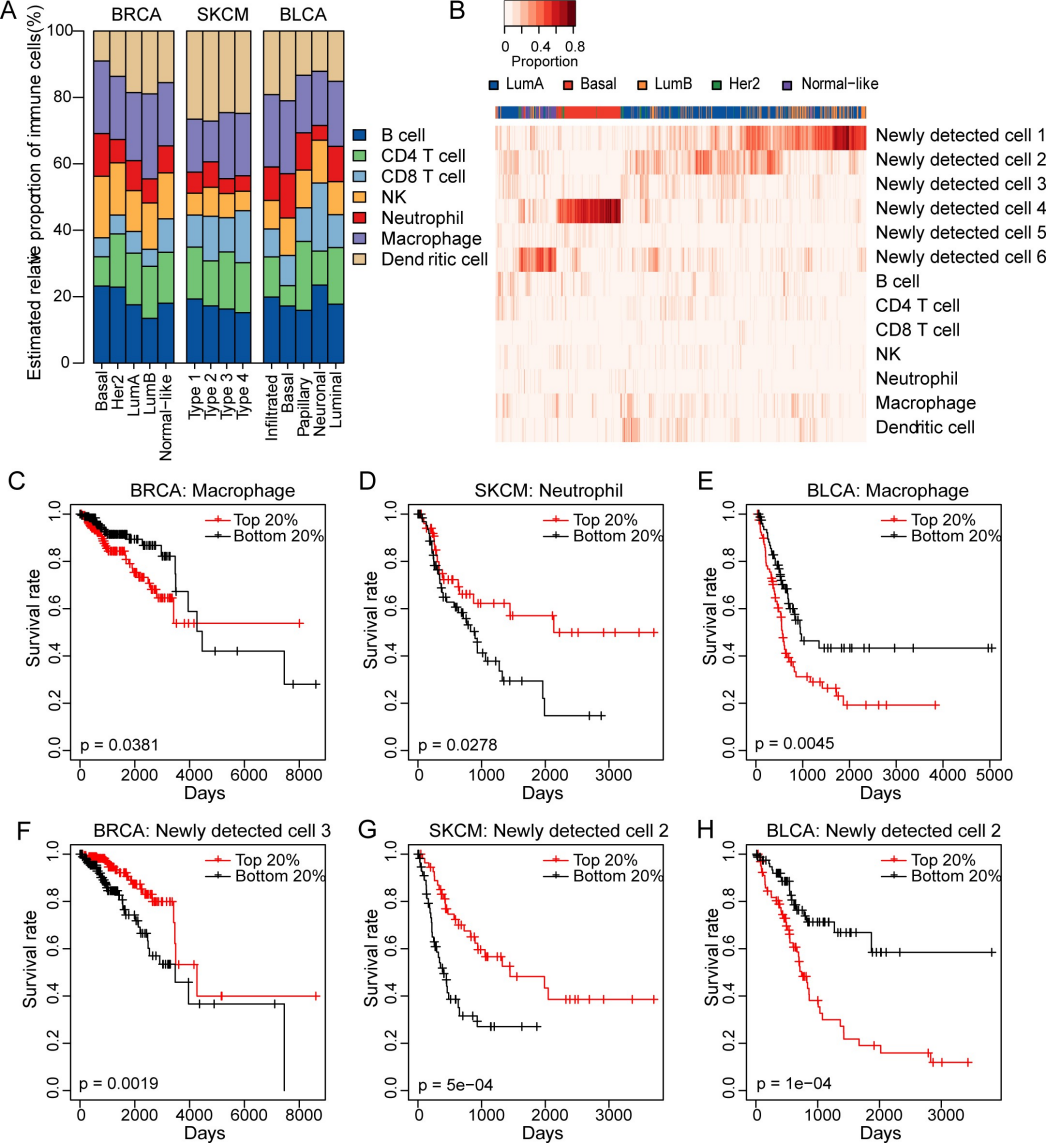
Application of PREDE to TCGA tumor samples. (A) Relative proportions of seven immune cell types in different subtypes of BRCA, SKCM, and BLCA. (B) Heatmap shows the absolute proportions of several types of cancer cells and immune cells in breast cancer samples. (C-H) Kaplan-Meier survival curves for BRCA stratified by abundances of infiltrated Macrophage (C) and newly detected cell 3 (F), SKCM stratified by abundances of neutrophil (D) and newly detected cell 2 (G), and BLCA stratified by abundances of Macrophage (E) and newly detected cell 2 (H). Patients with the top 20th percentile of immune/cancer cells were compared with those with the bottom 20th percentile. P-values are obtained by the Log-rank test.

Tumor tissues are mixtures of different cell types including mostly subclonal cancer cells as well as a fraction of infiltrating immune cells, stroma and blood vessel cells [30]. In this section, we further applied our method to TCGA tumor samples of three tumor types with the gene expressions of seven immune cells as partial reference. Based on these data, PREDE identified that the total numbers of cell types in BRCA, SKCM, and BLCA were 13, 10 and 12, respectively, according to the lowest AIC_c_ values. As expected, different subtypes of tumor samples showed distinct immune cell infiltrating patterns (Fig 7A). Macrophages account for the largest proportion of immune cells in all five subtypes of breast cancer and bladder cancer samples, which is consistent with previous experimental studies that high infiltration of tumor-associated macrophages is a hallmark of inflammatory breast cancers [31]. But the result for skin cutaneous melanoma was quite different, i.e., dendritic cells constituted the most part in SKCM samples, followed by macrophage and B cells. Interestingly, the proportion of CD8 T cells was significantly higher in Neuronal samples of bladder cancer compared with the other four subtypes, which may explain the best overall survival rate compared with other subtypes [32,33].

We then used proportions of all cell types to cluster breast cancer samples. All 980 breast cancer samples were categorized into the following five intrinsic subtypes, i.e., 508 Luminal A samples, 190 Luminal B samples, 78 HER2 samples, 169 Basal-like samples, and 35 Normal-like samples, based on gene expression profiles of PAM50 marker genes. Using the expression profiles of the 980 BRCA tumor samples and 7 immune cell types as the input of the PREDE, we obtained the proportion estimation of all cell types for each sample. The distance between two tumor samples is measured by the Bray-Curtis coefficient [34] between proportions of all cell types. We found that the Basal and Normal-like subtypes were well recognized by proportions of newly detected cell groups 4 and 6, separately (Fig 7B). Note that these newly detected cell types are not necessarily cancer cell types, but may be altered versions of known cell types, such as the infiltrating immune cells with new characters compared to those sorted from normal blood samples. This hypothesis could be tested by comparing single-cell expression profiles between certain types of infiltrating immune cells and their normal counterparts. To further examine the relationship between cell-type proportion and tumor subtype, we defined the heterogeneity score of each tumor sample as the Shannon index of its constituent cell type proportions. As shown in S9 Fig, different subtypes of tumor samples showed significant difference in heterogeneity score for all three cancer types (p = 2.6e-86, 3.6e-08 and 8.3e-11 for BRCA, SKCM and BLCA, respectively).

We next investigated whether the predicted proportion of cell type was associated with the survival of cancer patients (Fig 7C–7H). We first sorted tumor samples from one cancer type according to the estimated proportion of specific cell types (including known immune cells and estimated cancer cells), then calculated survival between the top 20% and the bottom 20% samples using Cox proportional hazards regression. We found that, for BRCA and BLCA, patients with a high level of macrophage infiltration show worse overall survival (p = 0.0381 and 0.0045) than those with low level of macrophage infiltration (Fig 7C and 7E), which indicates important roles of macrophage cells in prognosis and treatment of breast and bladder cancers. This result is also supported by two independent studies using immunohistochemistry experiments [35,36], i.e., larger numbers of CD68 macrophages were significantly associated with worse overall survival in breast cancer patients. Also, the meta-analysis showed that increased macrophage density was associated with poor prognosis in more than 80% of breast cancer cases [37]. In skin cancer, a higher level of neutrophil infiltration is associated with favorable survival (p = 0.0278, Fig 7D), consistent with the previous discovery by Li et al. [16]. Besides immune cells, we also found that the proportions of several newly detected cell types were significantly associated with survival rate of patients (Fig 7F–7H).

## Discussion

In this paper, we proposed PREDE, a partial-reference based deconvolution method for heterogeneous samples by integrating an iterative constraint quadratic programing algorithm into the NMF framework. Our approach generalized previously developed reference-based and reference-free deconvolution methods. We showed, through comprehensive simulations and real data analyses, that PREDE could recover expression profiles of unknown cell types and proportions of all cell types in mixed samples under a reasonable parameter setting. One major advantage of PREDE over existing methods is its ability to infer proportions of new cell types other than known references, which could be useful for downstream analyses. For example, for solid tumor tissues that consist of subclonal cancer cells and infiltrating immune cells, expression profiles of immune cells are usually available, but the subclonal cancer cells are largely unknown. We showed from real TCGA tumor samples that the proportions of newly detected cell types are closely associated with tumor subtypes, and are also good indicators of patient survival (Fig 7F–7H).

Despite its merit, our study still suffers from the following limitations. First, our method needs the number of cell components as input, which can be correctly inferred by minimizing the Akaike information criterion in the simulation study. However, for tumor mixture tissues, the problem was far more complicated because every two cells in tumor tissue can be different. Cells in a tumor tissue can be classified into different numbers of groups at different levels of similarity thresholds. In other words, all cells in a tumor tissue form a hierarchical structure where one can get any number of clusters depending on ‘similarity’ between cells within each group. Therefore, we encourage the users to try different *K*s in their applications, and to choose the *K* which yields the reasonably distinct decomposed profiles for downstream analysis. Second, our PREDE method, which detects the number of constitutional cell types based on AICc, is only applicable when rare populations are moderate in proportion (more than 7% according to our simulation). In addition, our method (as well as other deconvolution methods) fails to separate cell types that evolve on a continuum. It is our future work to integrate time-course data or to incorporate single-cell expression profiles as pseudo-time reference for more reliable deconvolution. Third, our method (as well as qprog and RF) assumes the error to be independently and identically Gaussian distributed across different genes, which may not hold for other types of biological data such as DNA methylation or RNA-seq counts data. So further attention should be paid on developing new methods free of such error assumption for partial reference-based deconvolution.

## Materials and methods

### Data preparation

We simulated three synthetic datasets to comprehensively evaluate our method. First, gene expression profiles of 91 lung cancer cell lines are downloaded from the Cancer Cell Line Encyclopedia database (CCLE, https://portals.broadinstitute.org/ccle) to generate mixed samples with different mixing proportions. Second, a benchmark dataset for cancer immunology study is generated by mixing 3 breast cancer cell lines from CCLE, 3 types of immune cells (including CD4 T cells, CD8 T cells and B cells) from GEO with accession number GSE22886 [38], and 2 primary breast epithelial cell lines (MCF10A and HMEC) with GEO accession number GSE101921 [39]. The total 8 cell types were quantile normalized and mixed into 100 tumor samples with proportions of tumor cells, immune cells, and normal epithelial cells to be roughly 3:1:1. Third, our method was further tested on the mixed RNA-seq data of three rat tissues (i.e., Brain, Lung and Liver) (GSE19830 [21]).

In addition, we employed gene expression data of peripheral blood mononuclear cells (PBMCs) of 20 samples (GSE65136) as well as the corresponding flow cytometry measurements [10] to benchmark PREDE with other existing methods.

Furthermore, for real data application, we downloaded level 3 gene expression data of all Breast invasive carcinoma (BRCA), Skin cutaneous melanoma (SKCM) and Bladder urothelial carcinoma (BLCA) samples from GDC data portal (https://gdc.nci.nih.gov). Expression profiles of seven immune cells (including B cells, CD4 T cells, CD8 T cells, NK, neutrophils, macrophages, and dendritic cells) are available from the Human Primary Cell Atlas (HPCA) database [40], which were used as reference for PREDE deconvolution. As suggested by [16], we used ComBat [41] remove the batch effect between the above TCGA RNA-seq data and HPCA microarray data for normalization. For further analysis, we also downloaded subtype and survival information of those tumor samples from GDC using TCGAquery_subtype and TCGAanalyze_survival functions in the R package TCGAbiolinks [42].

### Feature selection

In order to reduce computational cost, we selected a fixed number of genes which are most informative for deconvolution. Coefficient of variation (CV), a standardized measure of dispersion of a probability distribution, has been commonly used in feature selection for various high-throughput data [43,44]. In this study, we calculated the CV for each gene from the bulk gene expression matrix and selected top 1000 genes with the highest CVs as input features for PREDE.

### The PREDE model

The main workflow of PREDE algorithm is briefly illustrated in Fig 1. Given an *n*×*m* matrix *Y* as the expression profiles of *n* genes in *m* tumor samples, we assume that these tumor samples are mixtures of *K* cell types with different mixing proportions. Denote the basis matrix *W* as expression profiles for these *n* genes in *K* cell types, and the proportion matrix *H* as proportions of the *K* cell types in *m* samples. The observed data *Y* is assumed to be a linear combination of cell type-specific expression profiles, i.e., *Y* = *WH*+*ϵ*, where *ϵ* is an *n*×*m* error matrix. We aim to solve *W* and/or *H* from *Y*. If the basis matrix *W* is known, the problem is the typical reference-based deconvolution, which can be readily solved by the constrained linear regression (e.g., qprog [25]). If both the basis matrix *W* and the proportion matrix *H* are unknown, the problem is so-called reference-free deconvolution, which can be solved by the following NMF algorithm [19],

$$(\hat{W},\hat{H})=arg\;\underset{W,H}{\min}{\left\|Y-WH\right\|}_{F}^{2}.$$ (1)

However, in real clinical practice, only a fraction of the cell types in the tumor samples might be known. We denote the known portion of basis matrix *W* as *W*_1_, and unknown portion as *W*_2_, i.e., *W* = (*W*_1_, *W*_2_). Given expression matrix *Y* for all tumor samples and known reference matrix *W*_1_, we aim to infer the overall proportion matrix *H* and unknown basis matrix *W*_2_. Thus the above partial-reference based deconvolution problem can be formulized to

$$(\hat{W_{2}},\hat{H_{1}},\hat{H_{2}})={\mathrm{argmin}}_{W_{2},H_{1},H_{2}}{\left\|Y-(W_{1}W_{2})(\begin{array}{c} H_{1}\\ H_{2} \end{array})\right\|}_{F}^{2}=arg\;\underset{W_{2},H_{1},H_{2}}{\min}{\left\|Y-W_{1}H_{1}-W_{2}H_{2}\right\|}_{F}^{2}$$ (2)

subject to the following constrains: (a) nonnegativity of *W*_2_, *H*_1_ and *H*_2_; (b) column sum of *H*_1_ and *H*_2_ is less than 1.

We term the above problem (i.e., Eq (2)) as an iterative NMF model which could be solved through an iterative optimization strategy by developing a modified Quadratic Programming algorithm (Fig 1). More specifically,

- Start with a random initialization of *W*_2_;
- Fix *W*_1_ and estimate $H^{new}=\left(\begin{array}{c} H_{1}^{new}\\ H_{2}^{new} \end{array}\right)=\arg\;\underset{H_{1},H_{2}}{\min}{\left\|Y-W_{1}H_{1}-W_{2}H_{2}\right\|}_{F}^{2}$ subject to the constraints 0≤*h*_*ij*_≤1 and $\sum_{j=1}^{K}h_{ij}\leq 1$;
- Estimate $W_{2}^{new}=\arg\;\underset{W_{2}}{\min}{\left\|Y-W_{1}H_{1}^{new}-W_{2}H_{2}^{new}\right\|}_{F}^{2}$ subject to the constraints *w*_*ij*_≥0;
- Repeat steps (ii) and (iii) until convergence or a specific number of times.

### Determining the number of cell types using Akaike information criterion (AIC)

We used Akaike information criterion (AIC) [45] to determine the optimal number of cell types in mixture tumor samples. As a criterion widely used in statistical inference, AIC measures the goodness of fit of a model by balancing the tradeoff between loss function and model complexity. Since the number of samples is much fewer than the number of features, we used another version of AIC that is more suitable for small sample sizes (termed as AIC_c_). The basic formula of AIC_c_ is [46]:

$${AIC}_{c}=N\ln\left(\frac{SSR}{N}\right)+2p+\frac{2p(p+1)}{N-p-1}$$ (3)

where *N* is the sample size, *p* is the number of model parameters, and *SSR* is the sum of squared residuals between true and estimated gene expression profiles for all mixture samples. In the NMF framework (include PREDE as well), the samples size should be counted in the level of genes, i.e., *N* = *n*×*m*, and *p* = *K*(*n*+*m*)−*nK*_1_, where *n*, *m*, *K*, *K*_1_ are numbers of mixture samples, features, all cell types and known cell types, respectively. Compared with the original AIC, the AIC_c_ imposes a higher penalty when sample size is small, and approximates AIC when samples size increases. We calculated AIC_c_ for a reasonable range of potential cell type numbers (e.g., from 1 to 50) and the predicted optimal number of cell types were determined by the minimum AIC_c_ value.

### Deconvolution accuracy evaluation

We evaluated the performance of PREDE from the following two aspects: the estimation accuracy of basis expression matrix (*W*) and the estimation accuracy of cellular proportion matrix (*H*), which were assessed by Pearson correlation coefficient and the mean absolute error (MAE) between true and predicted cell type proportions.

## Supporting information

S1 Fig Correlation between true and predicted cell proportions using different methods. Pearson correlation of predicted cell proportions by five methods (A) at different levels of noise and (B) at different proportions of unknown cell types.
(TIF)

S2 Fig Performance of PREDE on different expression similarities of unknown cell type to known cell types. Accuracies of (A) proportion estimation and (B) profile estimation of our method based on ‘low similarity set’ and ‘high similarity set’.
(TIF)

S3 Fig Determining the number of cell types with rare population. AICc values at different numbers of *K* when proportion of rare cell types increases from 0.01 to 0.15.
(TIF)

S4 Fig Performance of PREDE for unknown rare cell type. Accuracies of (A) proportion and (B) profile estimations by PREDE and RF when proportion of rare cell type increase from 0.07 to 0.10.
(TIF)

S5 Fig Accuracy of proportion estimation for known rare cell type. Proportion estimations for rare cell type by four methods at (A) different proportions of the rare cell type and (B) noise ratios.
(TIF)

S6 Fig Accuracy of AIC_c_ in determining number of constitutional cell types. Blue line or red line show AIC_c_ values at different numbers of cell types when cancer cells or immune cells are unavailable, respectively.
(TIF)

S7 Fig Application of reference-free deconvolution method to cell line mixing data. Eight cell lines including three breast cancer cell lines, three immune cell lines and two normal cell lines were mixed together with proportions 60%, 20% and 20% respectively. (A-B) Accuracies of profile and proportion estimations when cancer cell lines are unknown; (C-D) Accuracies of profile and proportion estimations when immune cell lines are unknown.
(TIF)

S8 Fig Evaluation of PREDE, DeMixT and ISOpure on cancer, normal and immune cell line mixture data. Eight cell lines including 3 breast cancer cell lines, 3 immune cell lines, and 2 normal cell lines were mixed to simulate 100 tumor samples. Mean expression profiles of cancer cell lines, normal cell lines and immune cell lines were respectively treated as unknown cell components to validate all three methods using the rest cell lines as input. Estimations of (A) cellular proportion and (B) expression profile for the unknown cellular component by the three methods.
(TIF)

S9 Fig Shannon indexes of predicted proportions of cell types for each subtype of BRCA, SKCM and BLCA tumor samples. (TIF)
