# Supplementary figures and images for: Deconvolution of heterogeneous tumor samples using partial reference signals
Source: PLoS Comput Biol. 2020 Nov 30;16(11):e1008452. doi: 10.1371/journal.pcbi.1008452 (PMC7728196; doi:10.1371/journal.pcbi.1008452)

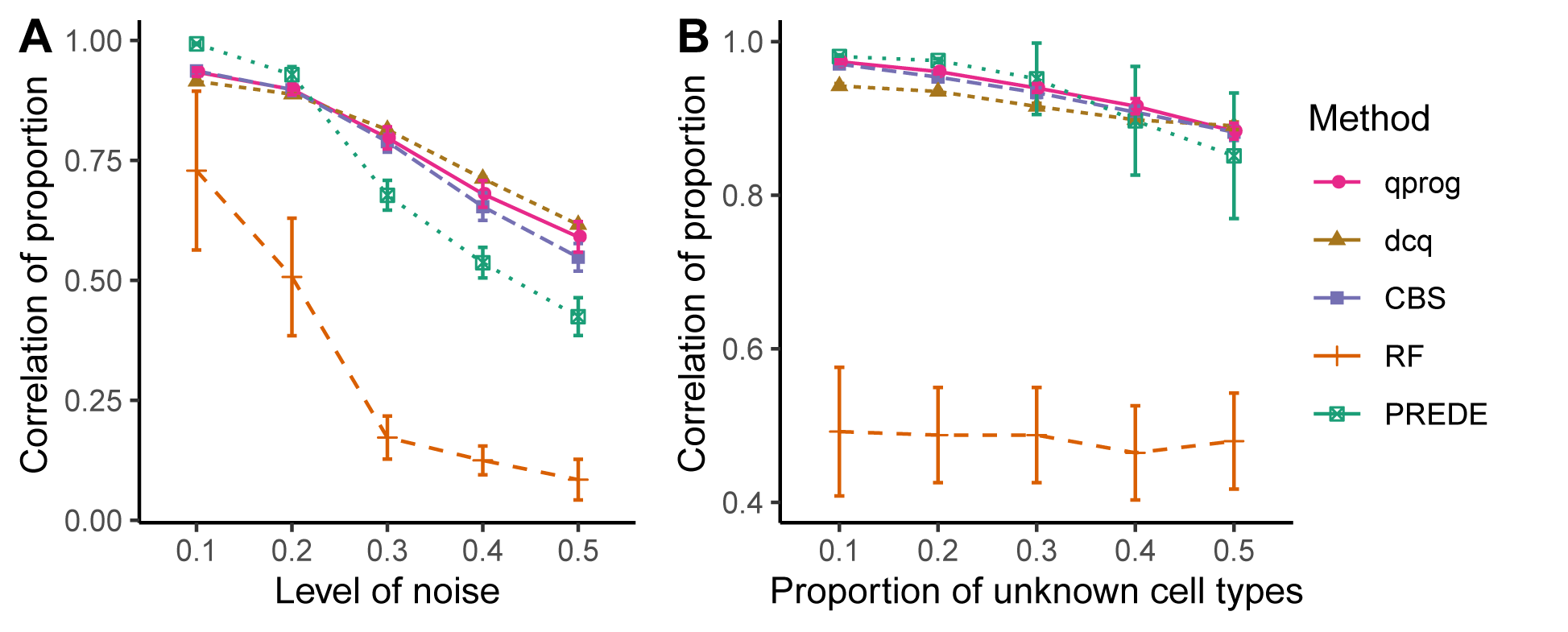

Supplement: S1 Fig — Pearson correlation of predicted cell proportions by five methods (A) at different levels of noise and (B) at different proportions of unknown cell types. (TIF) [file pcbi.1008452.s001.tif]

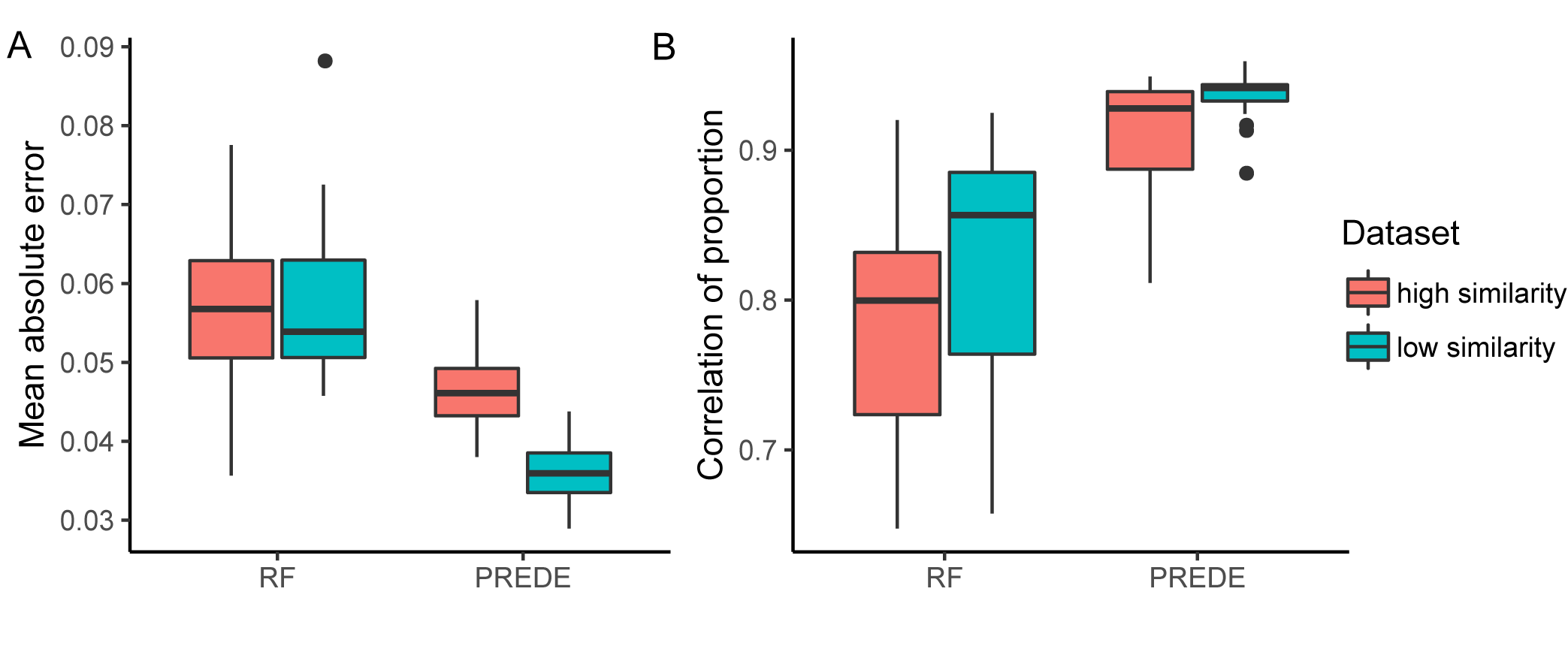

Supplement: S2 Fig — Accuracies of (A) proportion estimation and (B) profile estimation of our method based on ‘low similarity set’ and ‘high similarity set’. (TIF) [file pcbi.1008452.s002.tif]

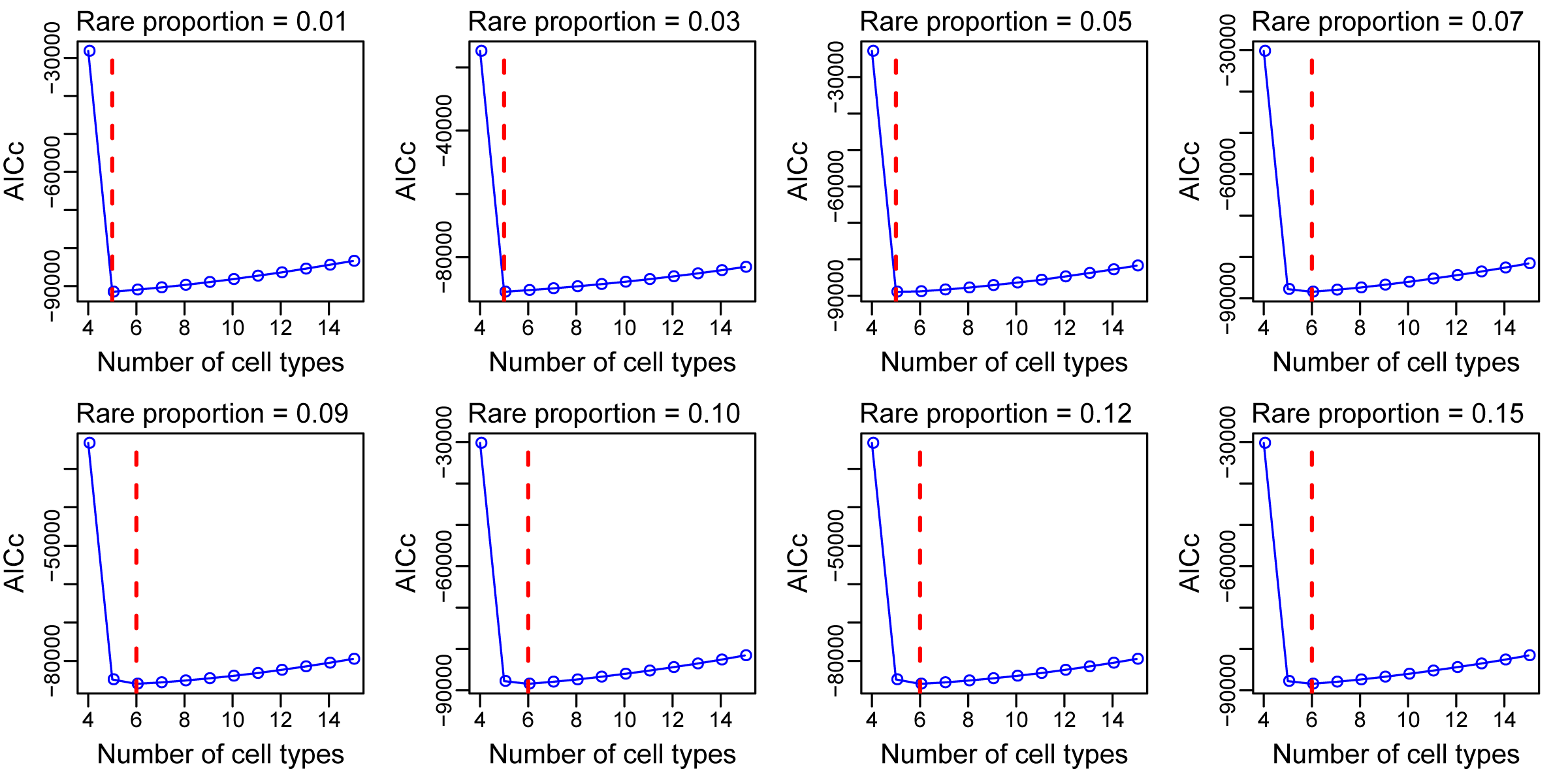

Supplement: S3 Fig — AICc values at different numbers of K when proportion of rare cell types increases from 0.01 to 0.15. (TIF) [file pcbi.1008452.s003.tif]

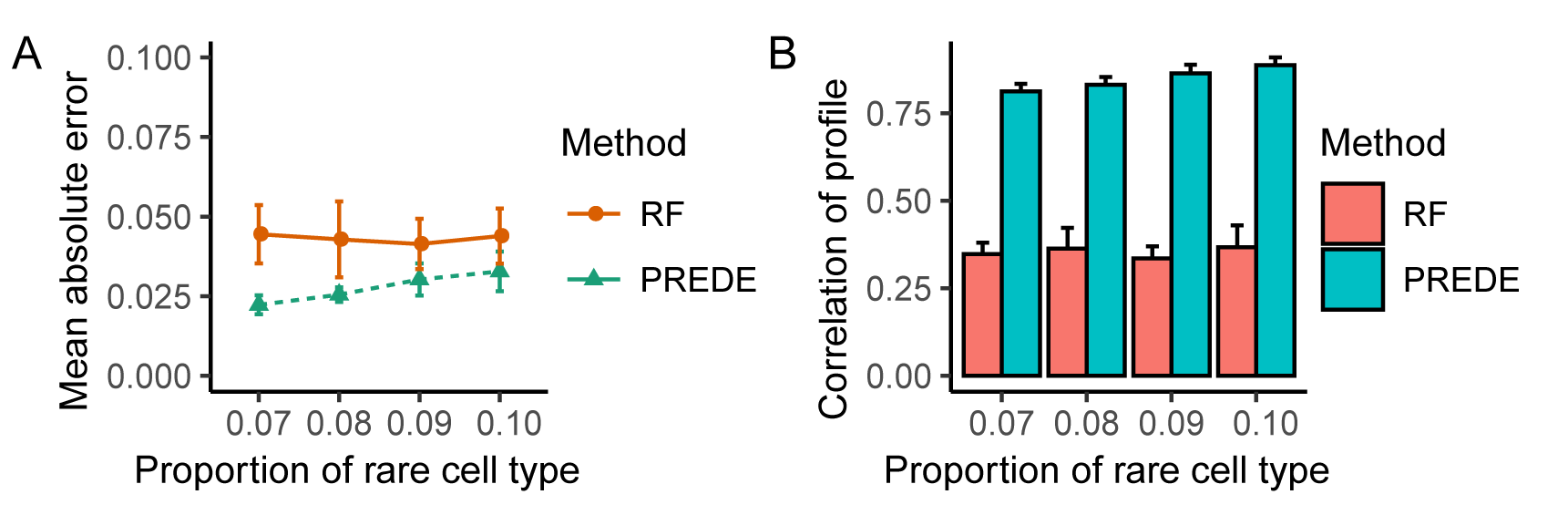

Supplement: S4 Fig — Accuracies of (A) proportion and (B) profile estimations by PREDE and RF when proportion of rare cell type increase from 0.07 to 0.10. (TIF) [file pcbi.1008452.s004.tif]

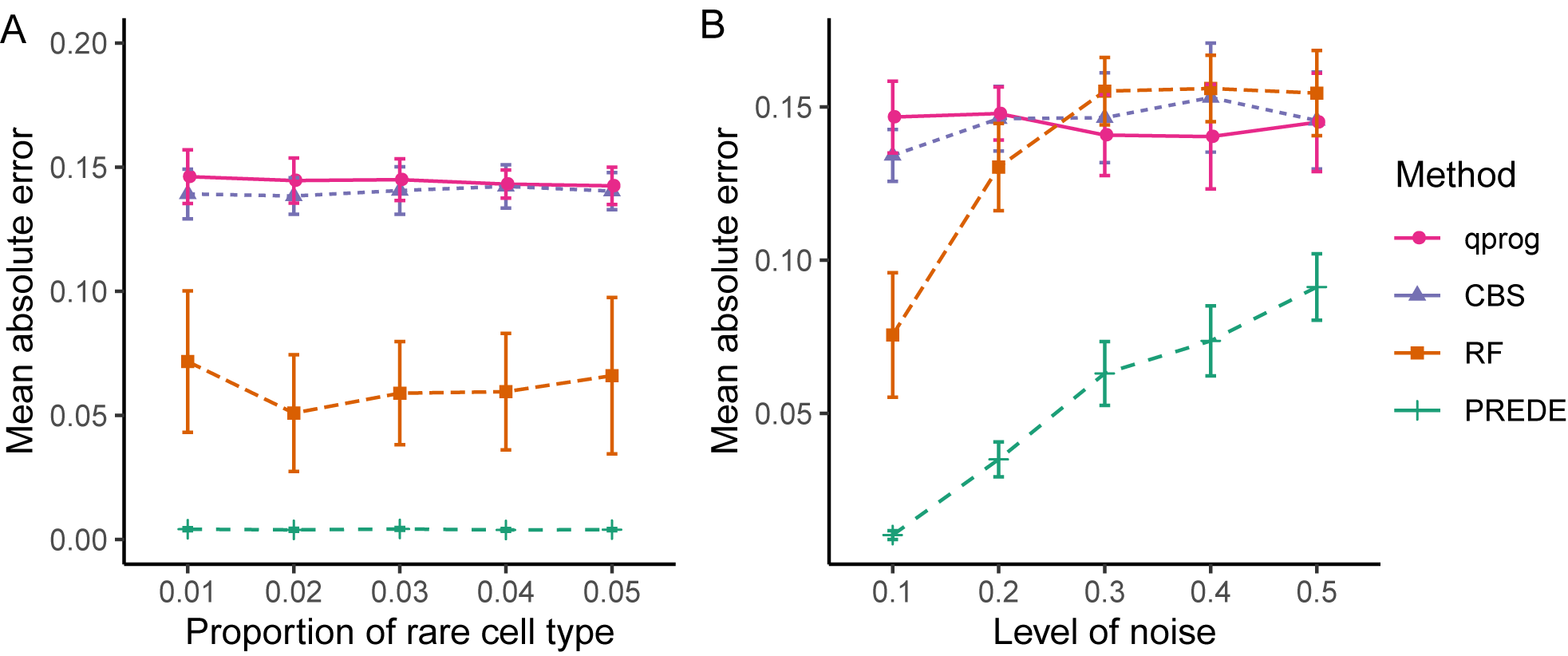

Supplement: S5 Fig — Proportion estimations for rare cell type by four methods at (A) different proportions of the rare cell type and (B) noise ratios. (TIF) [file pcbi.1008452.s005.tif]

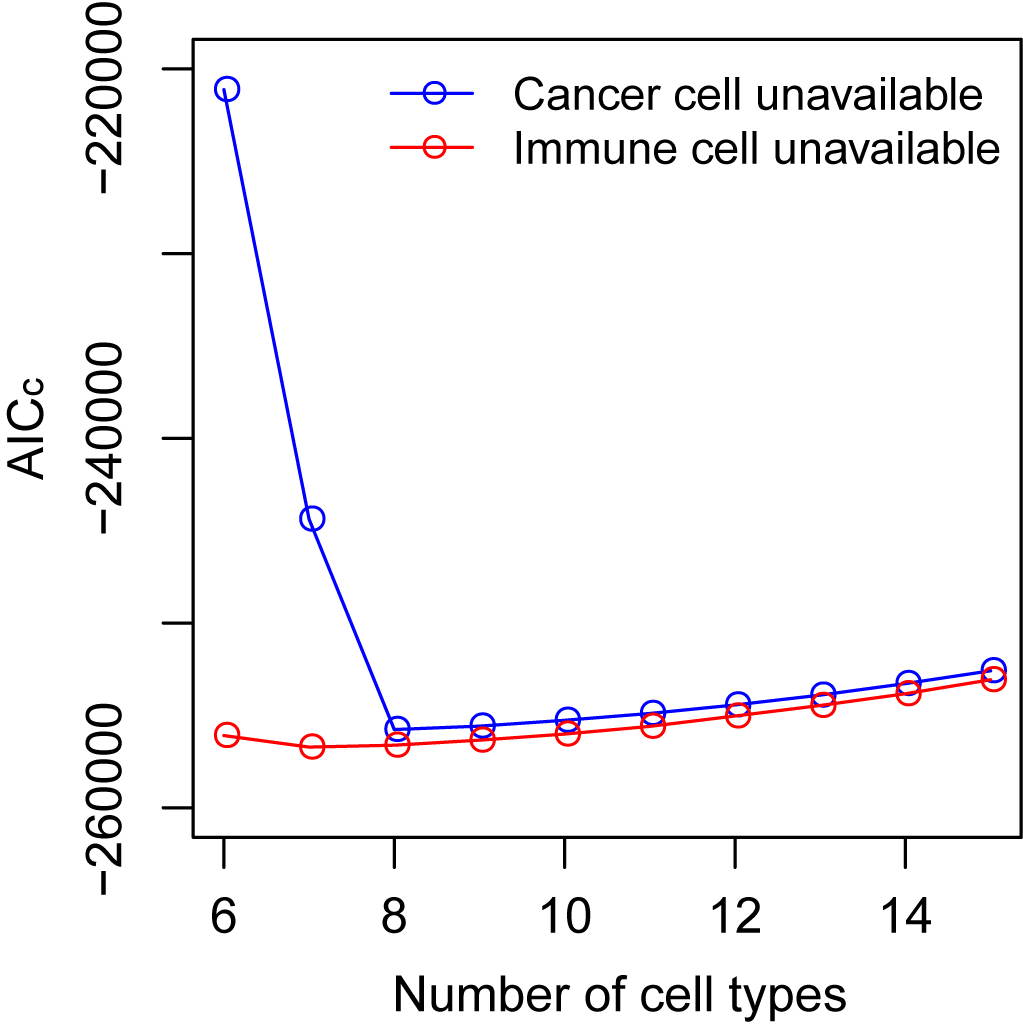

Supplement: S6 Fig — Blue line or red line show AICc values at different numbers of cell types when cancer cells or immune cells are unavailable, respectively. (TIF) [file pcbi.1008452.s006.tif]

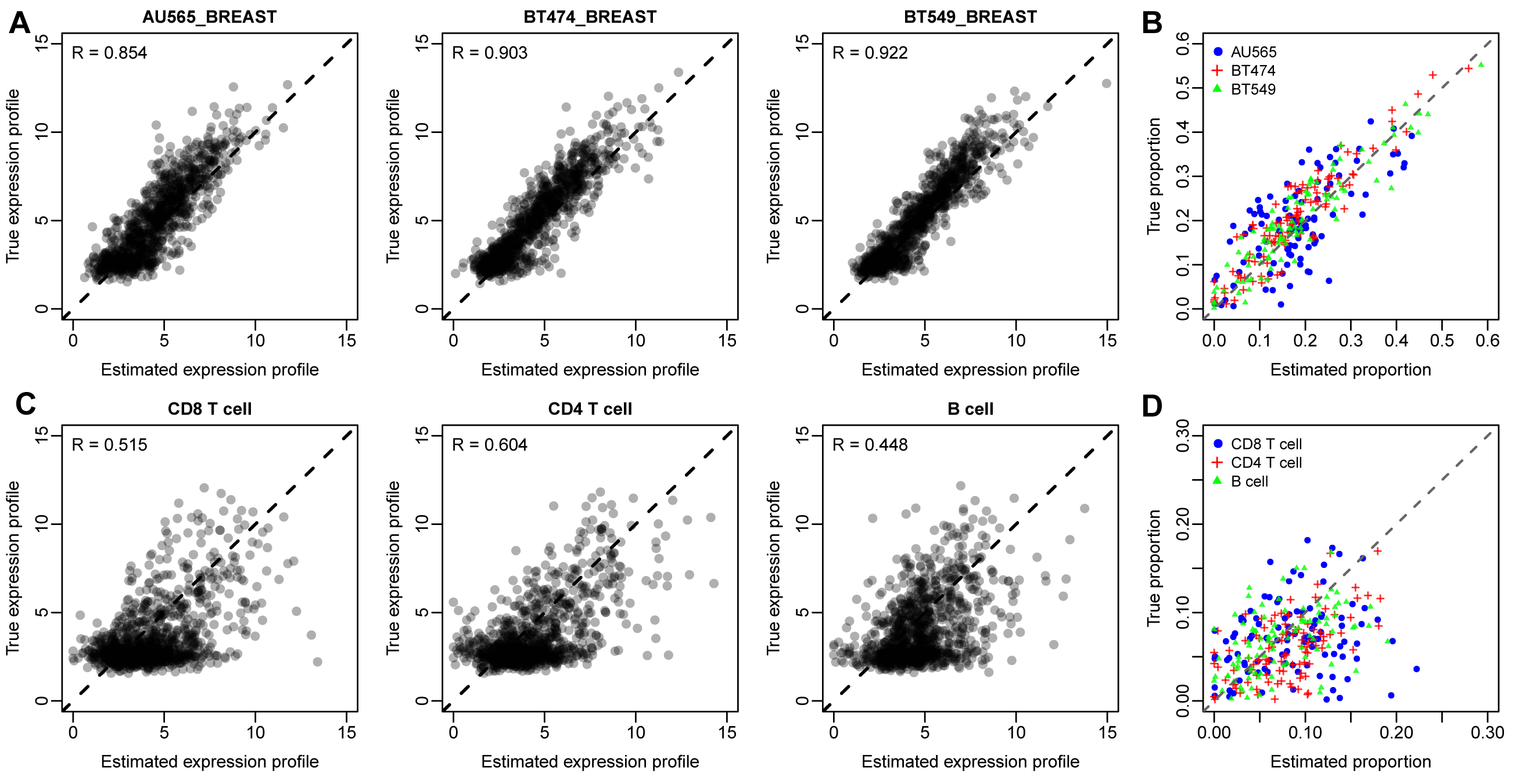

Supplement: S7 Fig — Eight cell lines including three breast cancer cell lines, three immune cell lines and two normal cell lines were mixed together with proportions 60%, 20% and 20% respectively. (A-B) Accuracies of profile and proportion estimations when cancer cell lines are unknown; (C-D) Accuracies of profile and proportion estimations when immune cell lines are unknown. (TIF) [file pcbi.1008452.s007.tif]

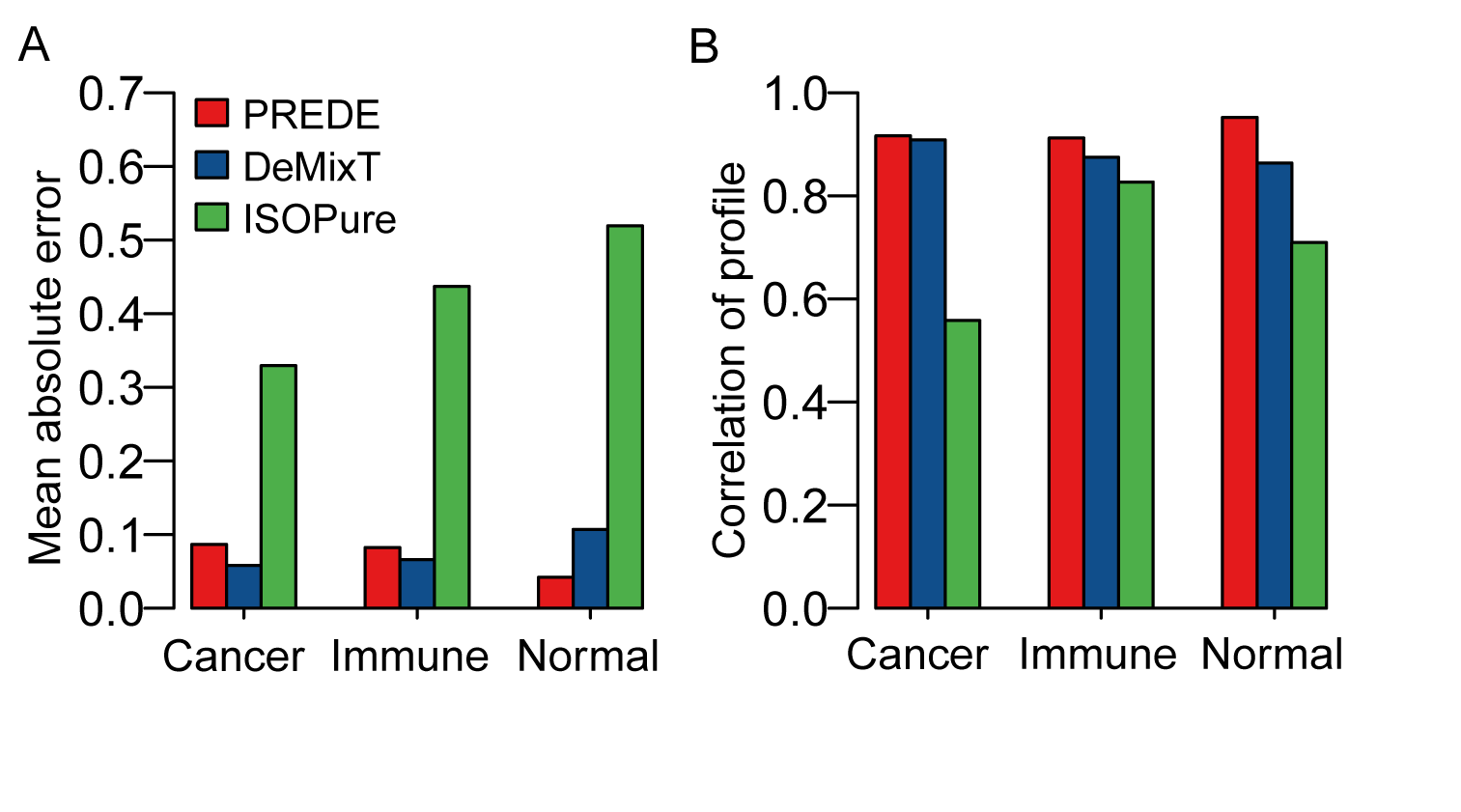

Supplement: S8 Fig — Eight cell lines including 3 breast cancer cell lines, 3 immune cell lines, and 2 normal cell lines were mixed to simulate 100 tumor samples. Mean expression profiles of cancer cell lines, normal cell lines and immune cell lines were respectively treated as unknown cell components to validate all three methods using the rest cell lines as input. Estimations of (A) cellular proportion and (B) expression profile for the unknown cellular component by the three methods. (TIF) [file pcbi.1008452.s008.tif]

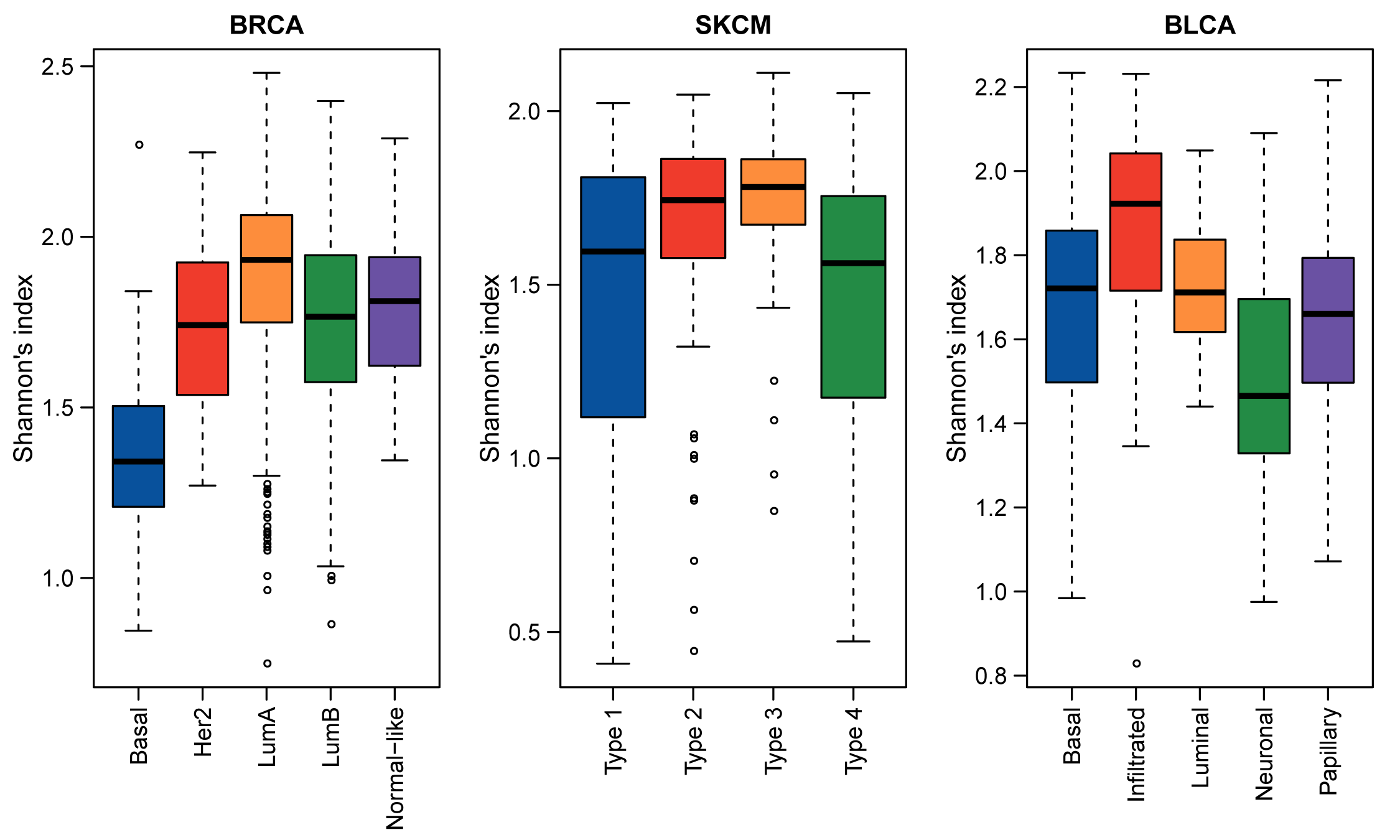

Supplement: S9 Fig — (TIF) [file pcbi.1008452.s009.tif]
